# Creation and Evaluation of a Preoperative Education Website for Hip and Knee Replacement Patients—A Pilot Study

**DOI:** 10.3390/medicina55020032

**Published:** 2019-01-30

**Authors:** Amelia Dayucos, Laverne Andrea French, Arpad Kelemen, Yulan Liang, Cecilia Sik Lanyi

**Affiliations:** 1 Department of Organizational Systems and Adult Health, School of Nursing, University of Maryland, Baltimore, MD 21201, USA; adayucos@lifebridgehealth.org (A.D.); lafrench@umaryland.edu (L.A.F.); kelemen@umaryland.edu (A.K.); 2 Department of Family and Community Health, School of Nursing, University of Maryland, Baltimore, MD 21201, USA; liang@umaryland.edu; 3 Department of Electrical Engineering and Information Systems, University of Pannonia, 8200 Veszprem, Hungary

**Keywords:** surgery, education, website, arthroplasty, usability

## Abstract

*Background and Objectives*: There is limited research on the question of whether web-based preoperative education can improve surgical patient outcomes. The purpose of this pilot study was to determine the usability, utility, and feasibility of a website created to increase engagement and improve the quality of the preoperative education that patients having hip and knee arthroplasty surgery receive. *Materials and Methods*: A website was created, and its appearance was designed with evidence-based “menu-driven” drop-downs to make the screen options age-appropriate to the patient population; the content was supported with video and PDFs of educational material, the same or similar to the usual education provided to patients. The patient-specific outcomes included qualitative data regarding patient knowledge, satisfaction, utilities, and usability. These objectives were assessed based on the perceived health website usability questionnaire online survey. Eighty patients who met inclusion criteria were recruited, ranging in age from 40 to 65 years old. Among them, 52.5% were female, 71.25% were scheduled for knee arthroplasty, and 28.75% hip arthroplasty. The patients were randomly assigned to the paper only or website education cohorts in a 50:50 ratio. However, only 19 from each cohort participated in the survey questionnaire. *Results and Conclusions*: We hypothesized that findings would show that patients receiving web-based education would feel more knowledgeable about their procedure, have less anxiety, and greater satisfaction with the addition of the website content; and that nurses would report that a website could conserve nursing time and resources. The study revealed no statistically significant differences between the cohorts, with an Alpha level set at 0.05. However, survey results showed that patients using the website rated self-perceived increase in knowledge, and their satisfaction in the time to find and review the information was higher than that of the paper-only cohort. The nursing survey revealed that website education improved workflow, efficiency, and patient education.

## 1. Introduction

It is estimated that 7 million Americans are currently living with hip or knee replacement, with the incidence of patients needing joint replacement skyrocketing. This will significantly impact the number of patients needing joint surgery education [1]. One study indicated the incidence of total joint surgery in the United States is now over 1 million procedures per year [2]. Knee and hip replacements are major operations that require tedious preparation to achieve a smooth process in the perioperative setting. As such, there is a growing need to deliver preoperative education to joint replacement patients. This is essential so that they can be aware of the requirements and obtain the knowledge they need prior to surgery. Likewise, surgical services and surgeons’ offices are busy environments that fast track patients’ preparation for surgery [3]. More specifically, orthopedic offices provide patient education through numerous means, such as e-mail, mails or letters, and short calls, to inform the patient about what to do and where to go, sometimes with minimal explanations.

Once these patients arrive in the preoperative centers, the nurses typically only spend 10 to 15 min to provide preoperative education to patients and families. This concludes with the patient leaving with a lot of preoperative documents, some of which are education-based. The process can be overwhelming, with the potential for causing anxiety [4]. Furthermore, the on-the-spot and quick preoperative education during the center visit does not allow patients time to absorb the information and ask relevant questions. To mitigate this problem, the creation of a preoperative education website for patients having hip and knee replacement is one way to educate patients and families at their convenience.

According to the literature, 74% of all U.S. adults utilize the Internet and 61% have looked for health or medical information on the Internet [5]. Accessing an online website prior to coming in to preoperative centers could allow patients to better obtain and absorb information. They would also be able to submit any questions they have while they are on the website and a preoperative team member could respond to their questions. Failing to fully address patient education needs could eventually become a patient safety issue. A systematic review of preoperative education for orthopedic patients showed a positive relationship between well-designed preoperative teachings and patients’ anxiety and knowledge levels [6]. Other studies have also shown that preoperative education plays an important role in improving patient outcomes and satisfaction with their surgical experiences in the perioperative setting [7,8].

Many prior studies reported the benefits of web-based preoperative education. For example, one study reported that the web-based education program in busy preoperative care areas can improve surgical patient education [9]. Another study demonstrated increased knowledge achieved with internet-based education compared with face-to-face education provided by a nurse in an ambulatory orthopedic surgery center [10]. A randomized trial revealed higher knowledge levels in patients utilizing computer-based education compared to face-to-face teaching in the office, thus enhancing the delivery of care [11]. This mode of educating the patient must also be specific to the needs of a particular patient population [12].

In this research project, we sought to create and evaluate usability, utility, and feasibility of a preoperative website for the education of patients having hip and knee replacements in order to augment knowledge. The hope was that creating a preoperative education website, an informatics-based quality improvement innovation, would enhance the patient education care process. Web-based preoperative teaching can better incorporate evidence-based research into this important aspect of clinical practice and patient education.

### 1.1. Aim of Study

The purpose of this pilot study is to determine the usability, utility, and feasibility of a website created to improve the quality of the preoperative education patients receive in preparation for hip and knee replacement surgery. This was accomplished by: (1) gauging patient satisfaction on knowledge acquired with the typical paper education forms; (2) developing a preoperative education website; (3) evaluating the usability and utility of the preoperative education website; and (4) assessing the preoperative education website with regard to its feasibility to the host facility. As to feasibility, the question is: Does providing online education to patients preoperatively help nursing workflow and increase nursing efficiency? Augmenting patient preoperative education with computer-based teaching has been shown to allow for more time for direct patient care and pre-surgical preparation [11].

### 1.2. Usability, Utility, and Feasibility

Website usability can be defined as the extent to which a website provides what the end-user is trying to accomplish [13]. It has also been described simply as “user-friendliness” [14]. In the context of web-based education for knee and hip replacement patients, some of the things to consider with regard to usability include: making sure the screens are not too busy, looking at ensuring that the tasks to maneuver around the website are not multistep processes, and, lastly, whether it improves the patient’s ability to seek and acquire their educational needs and does not make it more burdensome.

The concept of assessing utilization entails looking at how the patients perceived the benefits of preoperative education [15]. Did they feel that the website prepared them for surgery? Would they recommend it to a friend considering having arthroplasty surgery? Did the 24/7 available knowledge and links to nurse resources relieve some anxiety they had about the surgery? Knowledge deficit has been shown to be a possible contributor to anxiety prior to surgery [16]. In general, was it useful in providing a comprehensive single-source site for preoperative education, or did the patients feel they still had to go elsewhere to obtain instruction they felt they needed prior to surgery?

Some questions that would need to be answered in the affirmative from this study include: did the patients retain the information the same or better with the website versus the usual education methods such that there would be savings from decreasing the amount of paper to print the many learning materials? Was there increased adherence to family support planning and post-discharge instructions such that there would be a decrease in post-operative complications that would require readmission? Were less “live” nursing resources utilized for patient re-education such that nurses could now work more efficiently on other preoperative preparation? The benefits of a properly executed and effective preoperative patient educational intervention have been shown to result in improved psychological and physical well-being for patients undergoing surgery, leading to better outcomes [2].

### 1.3. Creation of the Website

#### 1.3.1. Choosing a Website Platform and Name

In choosing a website format, the team considered many website creation software products and websites. The choices were narrowed down to WordPress© and Wix©. The ultimate choice was Wix© due to the ease of the manipulation and customization of the site’s prebuilt templates. The company also provides Google analytics to users who purchase a domain name (or link their own to the site). The template design was chosen to be as simple as possible. This was felt to be important to obtaining data determining how many patients perused the site and may not considering the 50+ age group within which most hip or knee patients fall.

Our team designed and developed the website to ensure that the information was kept the same or similar to the education documents patients are presented with at their appointment in the Pre-Anesthesia Screening Services department (P.A.S.S.). A name was chosen for the site that could be easily remembered: www.jointsurgeryeducation.info/. Facility approval of the website was obtained before it went live.

#### 1.3.2. Description and Layout of the Website

In designing the website, the team kept the menu buttons at the top of the page, since this was a scrolling website, which is a more modern website design. Literature has indicated that seniors prefer menu-driven button options to navigate a web portal versus serial navigation [17]. Serial navigation is when the next most important information only becomes available after clicking on the initial option, link or button [18]. The default scrolling design was difficult to alter in Wix©. Therefore, we provided links on the home page to PDFs for the documents that patients would receive in P.A.S.S. Links that open up another window work better than “buttons” directing users to a pop-up and is optimal for mobile devices [18]. As this study was intended to compare the same information provided to patients, creating PDFs of the scanned documents for menu links was necessary to provide this parity of information. 

**Figure 1:**
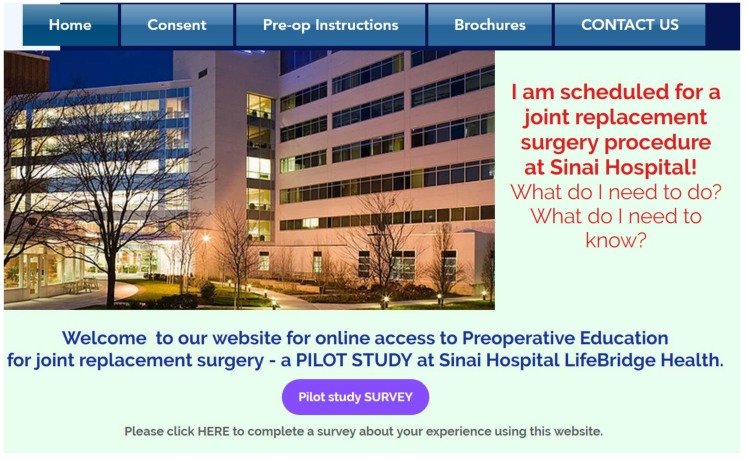
Screenshot of the Main Page of the Website.

The front page of the website contained the most critical information for the patients, hence the reason the front page was allowed to remain in rolling format. A video from the education class that patients attend was linked on that front page, as well as required preoperative preparation activities. The location and what to do and where to go on the day of surgery are also on the website’s front page. (A screen shot of a portion of the main page of the website can be found in Figure 1.)

#### 1.3.3. Content of the Website

The foundation and framework of the website content was based upon principles of patient empowerment through knowledge augmentation. The site contains preoperative information provided by one of the community hospitals in Maryland that follow anesthesia policies and protocol, orthopedic guidelines, and Joint Commission preoperative requirements for surgery. The website includes a checklist covering pre-operative testing requirements patients need to obtain before surgery. The checklist helps prevent delays and cancellations of surgery. It also lists fasting guidelines, medications to stop taking at least a week before surgery, things to bring on the day of surgery, and what to expect pre-operatively.

The website also includes videos about what to expect regarding joint replacement surgery and recovery. After viewing the videos, patients can ask questions via the site. These questions go directly to an email in-box for the P.A.S.S. nurses to answer. A phone number for the education class coordinator is available as another option for patients to call if questions arise after viewing the video. The brochures usually given by the community hospital in paper forms to patients during their preoperative visits (e.g., surgical site infections, fall prevention, anesthesia instructions, hand hygiene, MRSA infections, VRE infections, Sage wipes instructions, and nasal swab instructions) can be downloaded, printed, and shared directly on computers, tablets, mobile phones, using any modern web browser. Availability of these brochures on the Internet allows patients to read ahead of time and understand the importance of measures to prevent complications during and after surgery. Likewise, it is easier for the preoperative nurses to reinforce information in the brochure in a short period in the preoperative centers compared to giving these handouts during the visit and also conducting the educational teaching. On the main page, there are menu buttons to link to the informed consent, the purpose of the study, and biographies of the study team. The pilot survey questionnaires are embedded in the website and linked to Survey Monkey© for study purposes.

## 2. Materials and Methods

### 2.1. Study Settings, Sample, and Design

A prospective study was used to determine usability and utility of the online website, while a qualitative questionnaire was used to determine the feasibility aspect of the study. The study was conducted in a small community hospital in Baltimore, Maryland. The selected study sample was a convenience sample of two cohorts. The content of survey questionnaires was based on the Perceived Health Website Usability Questionnaire (PHWSUQ) for older adults [14]. 

The first cohort questionnaire was for patients who received the paper education in the preoperative center. The survey contained ten questions using a 7-point Likert scale (1 being Very Unsatisfied and 7 being Very Satisfied) assessing the following dimensions: satisfaction, ease of use, and utility [13]. The first three questions addressed the ease of use of paper educational material the patients received during preoperative visits. Questions 4–7 were about the degree of knowledge the patient acquired via the paper education as well as logic of organization of paper materials. Questions 8–10 asked about the usefulness of educational content in relation to decreasing patient anxiety and its utilization.

The second cohort survey questionnaire was for patients who accessed the preoperative website. The survey included fourteen questions utilizing the same 7-point Likert scale. The first five questions address the patients’ satisfaction of the preoperative website. The sixth to tenth questions involve the usability of the website in patients’ perspectives using a 7-point Likert scale (1 being Strongly Disagree to 7 being Strongly Agree). The rest of the questions measure the usefulness and utilization of the website information.

### 2.2. Data Collection

The primary research investigators conducted data collection and analysis from December 2016 to March 2017 for both cohorts. The method of data collection for website usability and utility were patient questionnaires through Survey Monkey©. Inclusion and exclusion criteria were used as guidelines in the selection of study subjects. The inclusion criteria of the cohorts included patients who were scheduled to undergo their first or second hip or knee replacement, can speak English, were between the ages of 40 and 65 years old, had no mental or severe physical handicap, and had Internet access and an e-mail address. 

The first cohort included those patients who would undergo hip and knee replacements with the same specified criteria and would show up in P.A.S.S. department for preoperative blood typing and screening. These patients received the usual preoperative educational pamphlets and face-to-face education by P.A.S.S. nurses. During the pilot study, the provision of preoperative education concluded with e-mail collection and verbal explanation of the purpose of e-mail request. These patients later received a survey link that took them to a Survey Monkey© questionnaire about the preoperative education documents they received on that day. 

The second cohort included those patients meeting the same criteria who also received access to the preoperative education website. The primary investigator sent e-mails to patients who provided an e-mail address during the office visit. The e-mail contained a link to the website, instructions, and the purpose of the study. Additionally, the primary investigator called the patients the day before the appointments in P.A.S.S. to follow-up on the utilization of the website. The website guided patients to different sections for information and videos about their procedure, and a survey link was embedded into the website for patients to fill out before leaving the website. The website link distribution process also involved disseminating a pamphlet that contained web link as patient visited the P.A.S.S. department before surgery. The pamphlet served as a follow-up for those patients who did not access the website at home. At the end of data collection for both cohorts, survey questionnaires were distributed to nurses in the P.A.S.S. department to address the feasibility of the preoperative website for the education of hip and knee arthroplasty patients.

### 2.3. Ethical Consideration

This pilot study is a quality improvement research project that meets the exemption criteria of organizational Institutional Review Board (IRB) with approval IRB #2400. The study protocols were submitted to the IRB board and met the IRB exemption criteria under 45 CFR 46.102(d), 21 CFR 50.3(c), and 21 CFR 56.102© as per institutional IRB review policies.

A short consent was embedded in each Survey Monkey© questionnaire informing the patients of the study purposes, that participation was voluntary, and reiterating anonymity in survey participation. No personal information was collected during the study, and patient e-mails were stored on an encrypted and password-protected computer onsite at the institution.

### 2.4. Description of Cohorts

As noted in Table 1, a total of forty (*n* = 40) patients were selected to participate in the 1st cohort survey questionnaires. Twenty-two (*n* = 22) of these participants were female, and eighteen (*n* = 18) were males. Twenty-eight (28) of these participants were scheduled for knee replacement while twelve (12) were having hip replacement. Nineteen (*n* = 19) of these patients participated in completing the survey questionnaire, which gave us a response rate of 47.5%.

Forty (*n* = 40) participants were also selected to participate in the 2nd cohort survey questionnaire. Twenty (*n* = 20) of the participants were male, and the other twenty (*n* = 20) were female. Twenty-nine (*n* = 29) participants were scheduled for knee replacement, while eleven (*n* = 11) were having a hip replacement. Nineteen (*n* = 19) of the selected patients participated in completing the online survey available in the website.

## 3. Data Analysis and Results

There are many ways to evaluate usability, such as heuristic evaluations, user testing in a lab setting and user surveys [14]. User survey was the chosen method for this study. For statistical analysis, Microsoft Excel data analytics tool was used to compute the results. The Alpha level was set to 0.05 for the statistical results. 

**Figure 2:**
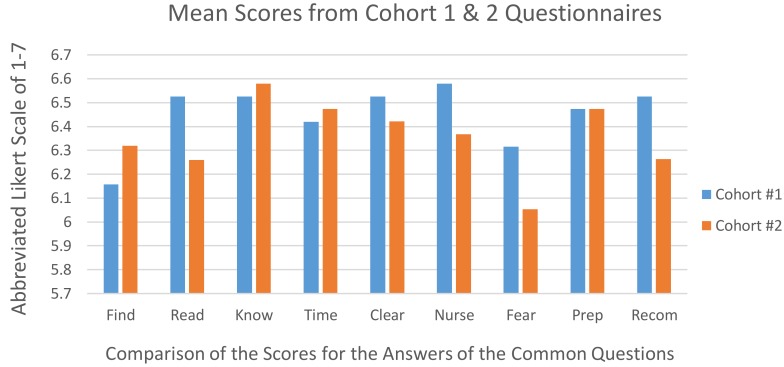
Bar graph displaying a comparison of the mean scores for the Cohorts.

As shown in Figure 2, patients’ perceptions of the education via the website were higher in three areas: the ability to find information they needed for their preoperative education, the knowledge they felt they gained from being able to also view their preoperative information via a website, and the website cohort patients also gave higher scores with respect to the time it took to peruse the materials.

In the present study, there is no statistically significant difference between the overall average scores of the website users and those patients receiving paper documentation. Also, both methods of information delivery (e.g., paper vs. paper and website) were both effective methods in increasing knowledge and engaging patient in their preoperative education on any major joint surgeries.

### 3.1. Usability Analysis

The usability of the new website was measured with the questions about how easy it was to find information, how easy it was to read it, how clear the information was, and the reasonableness of the time it took to peruse the site. The scores for how well the subjects felt that they could find specific information was higher for the website group, with a mean of 6.32 versus 6.16. The length of time that it took the study subjects to look over the materials via the website also received higher scores, 6.47 versus 6.42. These were the results when comparing the 9 questions in common for both cohorts.

When aggregating the grouping of the scores from the original 10 paper cohort questions and the original 14 questions from the website cohort’s survey, we looked for any significant differences for usability. One question that required an answer in the negative (in order to reflect a “positive” response) was removed, as these researchers realized that many patients likely answered it inaccurately, and it would have erroneously skewed the results in a downward trend. So, 13 questions from the website cohort were analyzed with independent samples *t*-tests for satisfaction, usability, and utility. In aggregate, the mean usability scores for the paper cohort is 6.48 and 6.35 for the web-based cohort, which was not statistically significant at the 0.05 level.

### 3.2. Utilization Analysis

The questions on the utility of the website had to do with how much it helped with easing fear or anxiety about surgery, how helpful the information was at increasing knowledge about and preparing for surgery, how helpful it was to be able to contact a nurse, and how likely the patient was to recommend the site to someone else. In assessing whether the subjects would find the website helpful in preparing for use, questions 9 and 10 for the paper education and questions 13 and 14 for the website revealed higher scores for the paper cohort than the website. 

In aggregate, the utility scores were 6.45 for paper education and 6.34 for web-based education. However, there is no statistical significance at the 0.05 level in terms of utility.

### 3.3. Knowledge

The subjects revealed scores of whether they increased in knowledge per question #4 for cohort one (mean 6.53) and question #6 for cohort two (mean 6.58). The website group scored their perceived knowledge increase higher. Although statistically insignificant, this is an important area of patient education to assess, as it indicates how much information the patient feels they received through the education provided. This is a critical quality indicator that has been linked to unanticipated post-surgical readmissions. The rate of surgical site infections is one of the patient safety indicators that healthcare regulators scrutinize. Education on how to prevent surgical site infections addresses this indicator. A website where the patients feel they have been provided superior knowledge on the subject of patient preparation for surgery, where, for instance, skin cleansing in preparation for a surgical wound is taught, are relevant to hospital quality statistics.

Additionally, education on anesthesia guidelines prior to surgery is relevant knowledge to prevent delays and cancellation of cases on the day of surgery. Preventing delays and cancellation of cases in the operating room is a financially-driven effort since it can affect Operating Room revenue as well as the organizational expense and ultimately patient costs. Preparation for surgery and knowledge of the upcoming procedure both scored higher for the website-enhanced education and this is a relevant factor for patient safety. This information in this pilot study is a promising sign for future studies as it shows that both delivery methods are satisfactory to patients.

### 3.4. Satisfaction

The scores regarding the subjects’ feelings of satisfaction with the information provided on the website are also captured in questions 1–3 for cohort 1 (mean 6.44) and questions 1–5 for cohort 2 (mean 6.28). There was no statistically significant difference between the subjects’ scores at the 0.05 level. The questions that were collecting common data for each cohort are presented in Table 2 and Figure 2. Table 3 provides the full data for questions presented to the subjects. The actual Survey Monkey© results, with the means and standard deviations for all questions to both cohorts, are also presented in Table 4.

### 3.5. Feasibility of the Website to the P.A.S.S. Department

A qualitative feasibility study was conducted using a survey questionnaire distributed to the nurses in the P.A.S.S. department. In addressing this goal, the study team sought to determine whether the staff would experience a benefit to having the website, in terms of nursing efficiency and cost-containment, while delivering the same content of preoperative education. The objective of this assessment was also looking for whether the nurses felt that increasing accessibility of the education could have a positive impact on the patient. The survey contained five items asking direct questions answerable by yes or no. Provision for a comment section was available in each question. A total of thirteen (*n* = 13) nurses participated in the survey. The first question asked the nurses if the preoperative website for hip or knee replacement helped expedite the process of the preoperative education they provided to patient visits. All thirteen (*n* = 13) nurses answered in the positive. A few commented that the website allows the patient to receive information early and to think about it ahead of time. One nurse mentioned that if the patient understands the preoperative education ahead of time, it will save the nurse’s time in explaining the whole preoperative process and teaching. 

The second question was about decreasing the amount of paper distributed to patients during the P.A.S.S. visit. Every participant’s response was affirmative that preoperative website would decrease paper trail process.

The third question queried the nurses on the capability of the website to aid in re-enforcing patient education preoperatively versus the paper education alone. The entire group of participants agreed that the website could be a positive re-enforcement of preoperative education to patients. A few nurses remarked that the website allows the patient to read the materials in advance and reinforcement would be easier as they come to preoperative centers.

The fourth question asked the nurses if the website would help educate family members and caregivers of patients in a convenient way. All thirteen (*n* = 13) nurses agreed that the availability of the website is another medium for family and caregivers to learn about the patient surgery. The nurses commented that the department conducts a Joint Effort Class for two to three hours requiring patients, family members, and caregivers to attend. The online availability of the Joint Effort Class video allows the patient, family, and caregivers to learn about the patient surgery in a convenient and easier way versus arranging for coming onsite to the hospital for 2–3 h.

The last question was about the relationship of the website’s availability and adherence to family support. All participants in the survey agreed that the website could help increase the adherence of household support. Few participants commented that families or caregivers who cannot accompany the patient during their preoperative center visit would not be able to listen to the education provided by the nurses during the visit. However, with the availability of the website, the preoperative education could also be viewed by family members and caregivers at their convenience. Also, with the availability of interactive e-mails and phone numbers, caregivers’ questions can be addressed in an easier way. Thus, family and caregivers can provide the support needed by the patient. Overall result, the nursing staff believed that the use of the website improved nursing workflow, efficiency, and patient education.

The full list of questions posed to the nurses can be found in Table 5. Table 6 shows the Nurse Survey Questionnaires on Feasibility. 

### 3.6. Accessibility Testing

The last test was the accessibility testing. 

In 1999, the World Wide Web Consortium published the Web Content Accessibility Guidelines WCAG 1.0 [19] and in 2008 the Web Content Accessibility Guidelines WCAG 2.0 was released [20]. For WCAG 2.0, the researchers recorded the number of known problems identified for A, AA, and AAA level guidelines. While WCAG 1.0 contains 14 guidelines with 62 checkpoints at 3 priority levels, the WCAG 2.0 has only 4 principles with 12 guidelines. Both levels of conformance have 3 levels: A, AA, AAA. Both provide technical advices.

The main principles and structure of WCAG 2.0:

- Principles—Top 4 principles.
- Guidelines—12 guidelines provide the basic goals.
- Success criteria—For each guideline testable success criteria are provided. Three levels of conformance are defined: A (lowest), AA, and AAA (highest).

**Figure 3:**
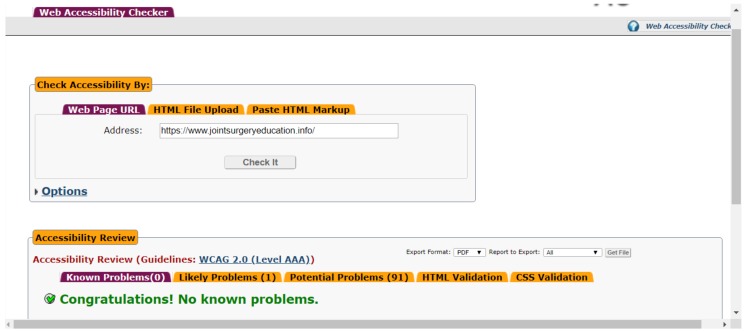
Screenshot of the AChecker automated tool (after the test, showing the results).

WCAG is part of a series of web accessibility guidelines published by the Web Accessibility Initiative (WAI) of the World Wide Web Consortium (W3C), the main international standards organization for the Internet. Legislations all over the world are based on the WCAG by W3C-WAI. The tests were performed by automatic checks using AChecker [21]. Figure 3 shows its results.

The AChecker did not find any “Known problems” at the strongest AAA conformance level. There is no doubt that the website was developed properly. It is easy to use and appropriate also for people with disabilities.

## 4. Discussion

The ability to provide patients with a website to obtain preoperative education is an efficient way to provide timely evidence-based information to patients. As mentioned above, studies have shown positive outcomes and patient satisfaction from this form of education [2,7,22,23]. As such, this study sought to expand this literature support to a local community hospital setting.

This being a pilot study, we were limited in having to utilize only the hospital approved education currently on hand. Thus, the creation of the website came with its challenges. It took a level of creativity to ensure that the structure of the website was as user-friendly as possible to the older adult population, while staying within the guidelines of providing the same or similar information that patients receive on paper. 

Currently, the preoperative center allocated 10 to 15 min of preoperative teaching to each patient coming in for preoperative work up. However, the patient has to attend a separate Joint Effort Class that usually last for two to three hours excluding the waiting time in the registration area and the preoperative unit, and the travel time. Hence, clinicians need innovative methods to deliver an effective preoperative education.

Findings from this study suggest that it would be beneficial to patients, family, caregivers, and providers to use a preoperative website as an additional preoperative intervention to improve patient education. Findings showed that both participants who received the paper education during their preoperative center visit and those participants who utilized the website were satisfied with the preoperative content of both methods. This means that both methods of preoperative education are effective ways of education. However, in terms of accessibility and convenience for patients and caregivers, the online website provides an advantage compared to paper education. One study reported that a web-based education program in the busy preoperative care area is an effective way of improving preoperative education [9].

Given that this was a first-of-its-kind study at this facility, having patients evaluating a website, there was some coaxing in order to prompt them to fill out the survey. It is our belief that in their desire to be supportive to the P.A.S.S. department nurses, they may have felt the need to provide good marks on both the paper education and the website scoring. Of the paper education cohort, 57% of the cohort gave marks of all 7’s (11 out of 19). For the website cohort, this number was only 26% (5 out of 19). This may be evidence of skewed results. 

With regard to feasibility of website-based education for patients, the results from the pilot study found that although there was no statistical significance of the study interventions, the survey results showed patients utilizing the website rated their self-perceived increase in knowledge higher and had more satisfaction in the time to find and review the information. This is evidence of a significant positive impact on the value of patient preoperative education delivered via a website. The optimistic opinions of P.A.S.S. nurses about the website education further support the benefits of the preoperative website to the organization. This quality improvement initiative would still be feasible to more efficiently deliver preoperative education once a few changes could be made to the study plan, when moving forward with future additional studies. With those changes, the researchers will likely eventually see a favorable increase of website education utilization of patients in the implementation of web-based preoperative education to older adults in the facility.

In summary, patient evaluations of the website were affirming and nursing opinions were positive. In a future study, when researchers have additional time to analyze results, sections allowing for free-texting comments by the patients could be added. This would further assist in the patients developing more individualized website education to meet their needs. In general, however, it does not appear that there were any major usability problems with the website. The “contact us” link to ask a question of a P.A.S.S. nurse portion of the website was not utilized by any patients. However, in larger studies of website utilization in the future, such a link will likely be utilized more often by patients. 

### 4.1. Limitations of the Study

Given that this study was a convenience sample and utilized a small sample size, generalizability of findings is limited. Future studies should focus on replicating this study with larger and different surgery patient samples. Moreover, the primary investigator noticed that engaging the older populations to utilize the website requires a lot of effort to “market” it to patients. It will remain difficult to capture the majority of the patients scheduled for hip and knee replacement surgery unless it became a required part of consent for surgery, at the time of the decision to proceed with surgery. One study found non-usage to be an issue when study parameters provided that the patients self-direct themselves to a website [24]. This means that further study should be conducted about patient engagement, specifically for the older adults pending surgery, and website utilization. 

This pilot study also only focused on knee and hip replacement patients and there is the possibility of comparing education via website for older patients having other surgeries. The result of that study can be used as a basis for creation and development of a preoperative websites in a more generalized area that includes all types of surgeries that are performed on older patients. That would require additional content and website development, unless the study could be conducted at different facilities that already have online website content, then the study could be targeted at obtaining data on the utilization of websites by older patients for preoperative education.

### 4.2. Opportunities for Future Research

Some other considerations to keep in mind for future studies might be additional evaluation of the patients who should participate in the study. We may want to include participants who have had surgery before (of any kind) with experience in receiving paper education in the past and also those who regularly utilize websites for healthcare education. This way we can better presume that our sample participants have had experience with both modes of learning. At the same time, we could also query additional questions such as the education level, household income, and employment status of the study types. Then lastly, it would also be helpful if we were able to pre-plan a longer lead time frame for the study to capture a larger population hip and knee surgery patients sooner to provide them the paper education first. In the study by Edward et al. [23], they captured almost 900 patients and provided preoperative education. Then, we could let time pass and then introduce them to the same education, but in the online format weeks or days prior to surgery. This would allow for paired sample survey testing.

## 5. Conclusions

The results of this pilot study indicate that website-based delivery of preoperative education for major joint replacement surgery is feasible and has similar patient satisfaction compared to traditional paper-based methods. Further research is needed to determine whether website-based education can promote more efficient and higher quality outcomes for joint replacement surgery.

## Figures and Tables

**Table 1:** Descriptive Characteristics and Usability and Utility Statistics of Paper Education (Usual Care) and Website Enhanced Education (Intervention) Cohorts (*n* = 80).

	Paper Education—Cohort #1	Paper & Website Education—Cohort #2
	(*n* = 40)	(*n* = 40)
**Age (years)**		
40–49	6	8
50–59	15	15
60–65	19	17
**Gender**	**Frequency (%)**	**Frequency (%)**
Male	18 (45)	20 (50)
Female	22 (55)	20 (50)
**Replacement**	**Frequency (%)**	**Frequency (%)**
Knee	28 (70)	29 (72.5)
Hip	12 (30)	11 (27.5)

**Table 2:** Questions common to both cohorts. Patient Evaluations on the Usability and Utility of the Website (showing the results of the independent samples *t*-test comparing only the nine “common” question items of the two cohorts).

	Paper (*n* = 19)	Website (*n* = 19)	*t-*test
Question Item (“Category name in Figure 2.”)	Mean (SD)	Mean (SD)	*p*
1. Ease of finding specific information. (“Find”)	6.16 (1.50)	6.32 (1.34)	0.74
2. Ease of reading the information given. (“Read”)	6.53 (0.75)	6.26 (1.33)	0.47
3. Reading the documents helped me improve my knowledge about my upcoming procedure. (“Know”)	6.53 (1.39)	6.58 (0.59)	0.88
4. I found the length of time needed to look over the materials/website appropriate. (“Time”)	6.42 (1.43)	6.47 (0.50)	0.88
5. The content of the materials/website provide clear information. (“Clear”)	6.53 (0.88)	6.42 (0.49)	0.66
6. I found information on how to contact the nurse for further questions helpful. (“Nurse”)	6.58 (0.88)	6.37 (0.48)	0.38
7. The content reduced my fears/anxiety about surgery. (“Fear”)	6.32 (1.03)	6.05 (0.89)	0.42
8. The content was useful in preparing me for surgery. (“Prep”)	6.47 (1.04)	6.47 (0.50)	1
9. I would recommend the materials/website to other people. (“Recom”)	6.53 (0.99)	6.26 (0.71)	0.37

Note. *T*-test significance for *p* = < 0.05.

**Table 3:** The Patient Cohort Questionnaires.

Preoperative Preparation and Teaching Website—Perceived Usability Patient Survey Questions for Total Arthroplasty Hip & Knee Patients * Adapted from: E.-S. Nahm et al./Development and Pilot-Testing of the PHWSUQ for Older Adults
*Questionnaire #1 (This was completed by the cohort who utilized usual P.A.S.S. education on PAPER.)* We would like to know your opinions about the paper documentation that you received for patient preparation and education on total knee and/or hip surgery (please circle your choice).
**Satisfaction**
1. Ease of finding specific information.
Very unsatisfied 1 2 3 4 5 6 7 Very satisfied
2. Ease of reading the information given.
Very unsatisfied 1 2 3 4 5 6 7 Very satisfied
3. Overall appearance of the pamphlets.
Very unsatisfied 1 2 3 4 5 6 7 Very satisfied
**Ease of Reading**
4. Reading the documents helped me improve my knowledge about my upcoming procedure.
Strongly disagree 1 2 3 4 5 6 7 Strongly agree
5. I found the length of time needed to look over the materials appropriate.
Strongly disagree 1 2 3 4 5 6 7 Strongly agree
6. The content of the materials provide clear information.
Strongly disagree 1 2 3 4 5 6 7 Strongly agree
**Usefulness**
7. I found information on how to contact the nurse for further questions helpful.
Strongly disagree 1 2 3 4 5 6 7 Strongly agree
8. The content of the preoperative education materials reduced my fears/anxiety about surgery.
Strongly disagree 1 2 3 4 5 6 7 Strongly agree
9. The content of the preoperative education materials is very useful in preparing me for my surgery.
Strongly disagree 1 2 3 4 5 6 7 Strongly agree
10. I would recommend these education materials to other people.
Strongly disagree 1 2 3 4 5 6 7 Strongly agree

**Table 4:** Excel Spreadsheets Exported from Survey Monkey.©.

Mean and standard deviation of patients rating on the * PHWSUQ for each item on the scale (n = 19)	
Item	Mean (SD)
1. Ease of finding specific information.	6.16 (1.50)
2. Ease of reading the information given.	6.53 (0.75)
3. Overall appearance of the pamphlets.	6.63 (0.67)
4. Reading the documents helped me improve my knowledge about my upcoming procedure.	6.53 (1.39)
5. I found the length of time needed to look over the materials appropriate.	6.42 (1.43)
6. The content of the materials provide clear information.	6.53 (0.88)
7. I found information on how to contact the nurse for further questions helpful.	6.58 (0.88)
8. The content of the preoperative education materials reduced my fears/anxiety about surgery.	6.32 (1.03)
9. The content of the preoperative education materials was useful in preparing me for my surgery.	6.47 (1.04)
10. I would recommend these education materials to other people.	6.53 (0.99)

**Table 5:** Nurse Survey Questionnaire and Data Table.

Nurse Survey Questionnaire
1. Do you think the availability of pre-operative website for hip and knee replacement patients’ help expedite the process of preoperative education in P.A.S.S. department? Yes: No: Comment:
2. Do you think the availability of the preoperative website for hip and knee replacement will decrease the amount of paper education provided to patients? Yes: No: Comment:
3. Do you think that with the availability of preoperative website for hip and knee replacement will help re-enforced patient preoperative education when they come-in to P.A.S.S. unit versus the paper documentation alone? Yes: No: Comment:
4. Do you think the preoperative website availability will help in educating family members and caregivers of patient in a convenient way? Yes: No: Comment:
5. Do you think the preoperative website availability for hip and knee replacement will increase adherence of family support? Yes: No: Comment:

**Table 6:** Nurse Survey Questionnaires on Feasibility.

	Total (*n* = 13)
Expedite nursing process (Y/N)	
Yes	13 (100%)
Decrease amount of paper education (Y/N)	
Yes	13 (100%)
Re-enforce education (Y/N)	
Yes	13 (100%)
Convenience to patient/family (Y/N)	
Yes	13 (100%)
Can increase adherence of family support (Y/N)	
Yes	13 (100%)
